# Successful local treatment for repeated hepatic recurrences of cholangiolocellular carcinoma: a report on a long-term survivor

**DOI:** 10.1186/s40792-017-0391-2

**Published:** 2017-12-02

**Authors:** Kentaro Shinohara, Tomoki Ebata, Yukihiro Yokoyama, Tsuyoshi Igami, Gen Sugawara, Takashi Mizuno, Junpei Yamaguchi, Yoshie Shimoyama, Shuichiro Shiina, Ryosuke Tateishi, Toru Arano, Masato Nagino

**Affiliations:** 1 Division of Surgical Oncology, Department of Surgery, Nagoya University Graduate School of Medicine, 65 Tsurumai-cho, Showa-ku, Nagoya, 466-8550 Japan; 2 Department of Pathology and Clinical Laboratories, Nagoya University Graduate School of Medicine, Nagoya, Japan; 3 Department of Gastroenterology, Juntendo University School of Medicine, Tokyo, Japan; 4 Department of Gastroenterology, Graduate School of Medicine, The University of Tokyo, Tokyo, Japan; 5 Department of Gastroenterology, Toshiba General Hospital, Tokyo, Japan

**Keywords:** Radiofrequency ablation, Intrahepatic recurrence, Intrahepatic cholangiocarcinoma, Cholangiolocellular carcinoma

## Abstract

**Background:**

Cholangiolocellular carcinoma (CoCC) is a rare liver tumor arising from the canals of Hering found between the cholangioles and interlobular bile ducts. Although morphologically CoCC mimics intrahepatic cholangiocarcinoma (ICC), CoCC exhibits a unique intermediate biologic behavior between hepatocellular carcinoma (HCC) and ICC. Curative resection is required for prolonged survival in patients with CoCC. However, effective therapy for postoperative hepatic recurrence has not yet been standardized.

**Case presentation:**

A 40-year-old man had an asymptomatic liver mass found during a regular medical examination. Contrast-enhanced computed tomography revealed a well-enhanced mass, 15 cm in diameter, in the right liver. He underwent right hemihepatectomy at a local hospital under the preoperative diagnosis of hepatocellular carcinoma. Pathologic examination confirmed a moderately differentiated tubular adenocarcinoma, leading to a diagnosis of ordinary ICC. Twelve months after surgery, he was referred to our hospital due to three hepatic recurrences in the left medial segment. He underwent partial hepatectomy for the recurrence, followed by adjuvant chemotherapy using gemcitabine alone. After the second hepatectomy, hepatic recurrences developed an additional seven times. The numbers and sizes of the recurrent tumors were very limited at each recurrence, satisfying the standard criteria for percutaneous radiofrequency ablation (RFA) for the treatment of HCC. All lesions were treated by percutaneous RFA, although this was an exceptional approach for ICC. He is now alive without evidence of disease 9.2 years after the first hepatectomy.

Because his clinical outcome was satisfactory and not compatible with the typical negative outcomes of ordinary ICC, we re-reviewed the histological findings of his tumor. The tumor was composed of small gland-forming cells proliferating in an anastomosing pattern; the cell membrane was strongly immunoreactive for epithelial membrane antigen. These findings were in accordance with the typical features of CoCC, revising his final diagnosis from ICC to CoCC.

**Conclusions:**

This case report demonstrates a satisfactory outcome using repeated local treatments, such as hepatectomy and RFA, for hepatic recurrences of CoCC, suggesting that a localized treatment approach can be considered to be a therapeutic option. We should be careful in making a definitive diagnosis of ICC and ruling out CoCC because the diagnosis potentially dictates the treatment strategy for recurrences.

## Background

Cholangiolocellular carcinoma (CoCC) is a rare primary liver tumor and was first described by Steiner et al. in 1959 [1]. This tumor is histologically characterized by small gland-forming tumor cells with variable extents of fibrous stroma. The morphologic features of CoCC resemble those of the canals of Hering, intervening between the cholangioles and interlobular bile ducts. However, until recently, this disease had been categorized as a variant of ordinary intrahepatic cholangiocarcinoma (ICC) [2]. Theise et al. observed that the canals of Hering potentially diverge to hepatocytes and cholangiocytes [3]. Thus, CoCC has an intermediate tumor biology between hepatocellular carcinoma (HCC) and ICC [4] and is considered to be an independent disease [5].

It is well known that CoCC has favorable survival outcomes after curative resection [6]. However, because of the rarity of CoCC, clinical outcomes in patients who relapse remain poorly understood. Herein, we describe a long-term survivor who had frequent recurrences of CoCC that were treated by hepatectomy and radiofrequency ablation (RFA). This is the first report showing the potential utility of RFA for recurrent CoCC.

## Case presentation

**Fig. 1:**
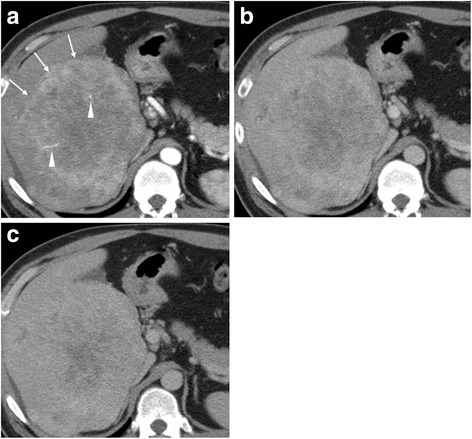
Computed tomography images. **a** Arterial-phase scan shows a peripherally well-enhanced tumor in the right liver (*arrow*) with vessel penetration (*arrow head*). Retention of the contrast media in the lesion is observed **b** from portal phase **c** to delayed phase

**Fig. 2:**
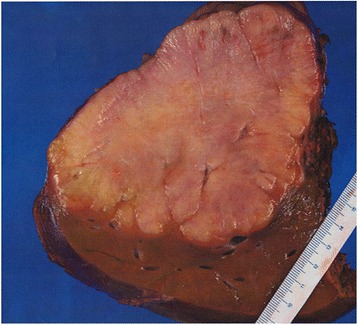
Gross finding of the primary lesion. Cut surface of the tumor shows a yellowish nodule 13 cm in diameter

A 40-year-old Japanese man presented to a local hospital for evaluation of an asymptomatic liver mass detected during a regular medical examination. He had no medical history of chronic hepatitis or fatty liver. Serum tumor markers were increased; the patient’s protein induced by vitamin K absence or antagonist-II (PIVKA-II) level was 2737 mAU/ml, and his alpha-fetoprotein (AFP) level was 23,054 ng/ml. The patient’s serum carbohydrate antigen 19-9 (CA19-9) level was not measured. Contrast-enhanced CT revealed a well-enhanced mass, 15 cm in diameter, in the right liver without extrahepatic lesion (Fig. 1). He underwent right hemihepatectomy without lymph node dissection under the preoperative diagnosis of HCC (Fig. 2). Histologically, a moderately differentiated tubular adenocarcinoma was found, leading to a pathologic diagnosis of ICC. According to the American Joint Committee on Cancer (7th edition) [7], this tumor was classified as pT1NXM0 and R0 resection was achieved.

**Fig. 3:**
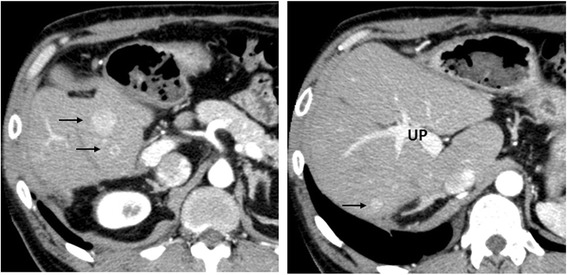
Computed tomography images of the initial recurrent lesions. Three well-enhanced masses in segment 4 are observed (*arrow*). UP umbilical portion of the left portal vein

An abdominal CT performed 12 months after surgery revealed three hyper vascular tumors in the left medial segment (S4), the size of which ranged from 8 to 26 mm (Fig. 3). He was referred to our hospital, and a partial hepatectomy of S4 was performed. The liver transection margin was positive (R1 resection) because one tumor was adjacent to the umbilical portion of the left portal vein. The operation time was 337 min, and the blood loss was 637 ml. He had a satisfactory postoperative course and was discharged on postoperative day 14. Pathologically, the recurrent tumors were similar to the primary lesion, leading to a diagnosis of recurrent ICC.

**Fig. 4:**
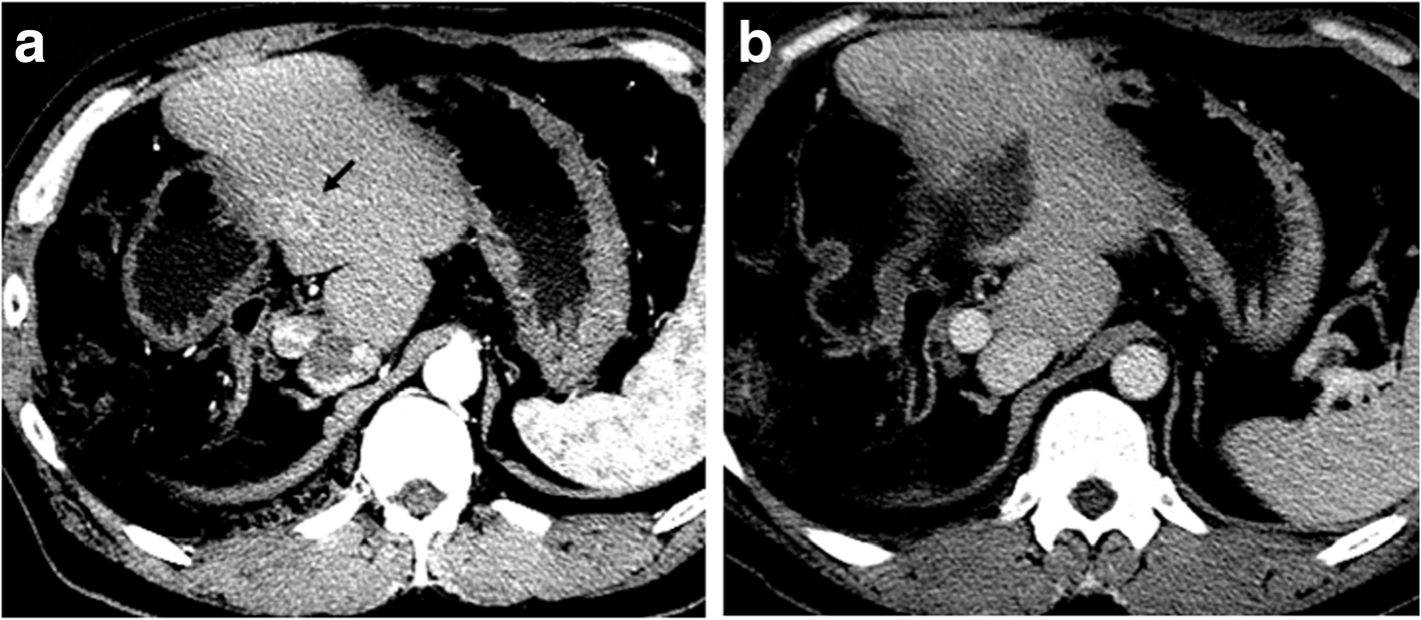
Computed tomography images of a recurrent lesion treated with radio frequency ablation. **a** A peripherally well-enhanced tumor is seen in segment 3 (*arrow*). **b** The index tumor is completely ablated with radio frequency ablation

The patient received adjuvant chemotherapy with gemcitabine alone (the sole regimen for biliary tract cancer that the Japanese national insurance system allowed at the time) for 10 months after the second surgery. However, the follow-up CT revealed a 15-mm hypervascular tumor in the left lateral superior segment (S2), which was compatible with a recurrence. As a result, he was then referred to another hospital for percutaneous RFA. Abdominal ultrasound at the referral hospital showed another 6-mm tumor in the remnant segment 4. Both lesions were successfully treated by ultrasonography-guided percutaneous RFA (Cool-Tips; RF Ablation System, Covidien, Boulder, CO) with a sufficient safety margin. Following the procedure, he underwent gadoxetic acid-enhanced magnetic resonance imaging (EOB-MRI) every 1 to 2 months for surveillance. In total, he developed hepatic recurrences an additional seven times following the second hepatectomy, and a total of 10 recurrent tumors in all were treated by percutaneous RFA (Fig. 4; Table 1). He is now alive without evidence of disease 9.2 years after the first hepatectomy and 4.7 years after the last RFA.

**Table 1:** Intrahepatic recurrence details

Recurrence event	Interval after primary hepatectomy (months)	Recurrence site (size)	Treatment	Serum CA 19-9 level (U/ml)	Serum CEA level (ng/ml)
1	12	S4 (8 mm), S4 (9 mm), S4 (26 mm)	S4 partial resection	32	2.8
2	22	S2 (15 mm), S4 (6 mm)	RFA	25	2.9
3	26	S2 (22 mm)	RFA	29	2.9
4	34	S1 (16 mm), S3 (10 mm)	RFA	35	3.4
5	36	S1 (10 mm)	RFA	29	2.7
6	41	S3 (7 mm), S3 (7 mm)	RFA	29	3.6
7	43	S3 (10 mm)	RFA	26	2.9
8	55	S2 (12 mm)	RFA	32	3.8

*RFA* radiofrequency ablation, *CA 19-9* carbohydrate antigen 19-9, *CEA* carcinoembryonic antigen

**Fig. 5:**
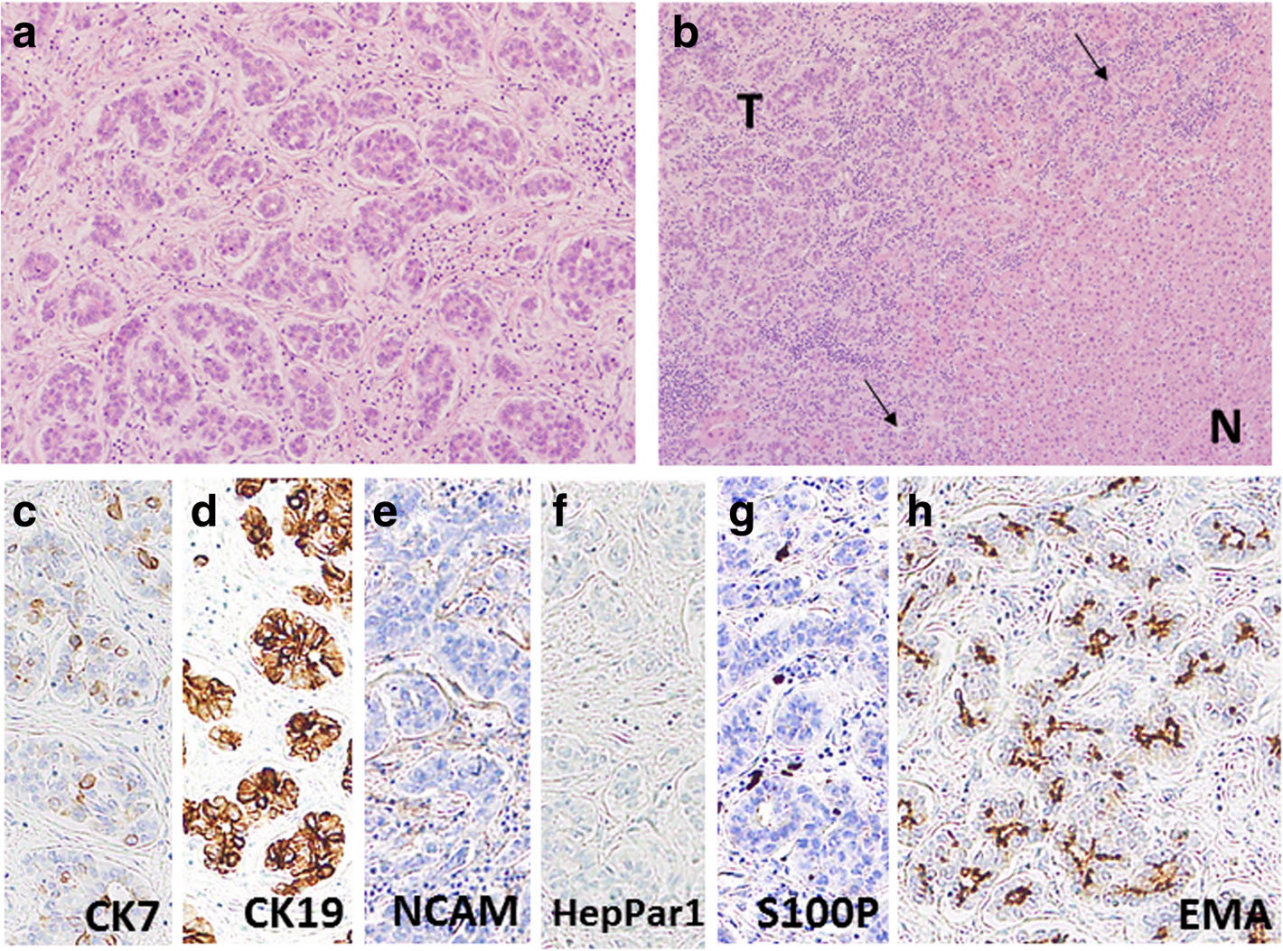
Histological findings of the recurrent lesion. **a** Small gland-forming cells proliferate in an anastomosing pattern with abundant fibrous stroma (HE staining, ×200). **b** The tumor cells (*T*) proliferate as they replace the noncancerous liver parenchyma (*N*) without tumor capsule (*arrow*) (HE staining, ×100). **c**–**g** Immunohistochemical studies show that the tumor cells are positive for cytokeratin (CK) 7 and CK19 and negative for neural cell adhesion (NCAM), HepPar1, and S100P. **h** Epithelial membrane antigen (EMA) stain shows positivity at the apical membranous areas of the ducts

Because his clinical course was not compatible with the usual negative course observed in patients with postoperative liver metastasis of ordinary ICC, we re-reviewed the histological findings of the initial recurrent lesions. The histological slides of the primary tumor were not available. Surprisingly, several specific features of CoCC were found: small-sized gland formation, proliferation in an anastomosing pattern, gradual transition to naive hepatocytes, and no mucin production (Fig. 4), while the portal vein area was not found within the tumor. The tumor contained neither ICC-like nor HCC-like areas. Immunohistochemically, the tumor cells were positive for cytokeratin 7 and 19 and negative for neural cell adhesion molecule (NCAM), HepPar1, and S100P. Epithelial membrane antigen (EMA) was strongly positive at the apical membrane of the tumor cells (Fig. 5). All these findings, except for negative immunoreaction of NCAM, strongly supported a revision of his initial diagnosis from ICC to CoCC.

### Discussion

CoCC shows a variable histological morphology depending on the extent of the associated ICC/HCC components. Such an inherent histological heterogeneity potentially complicates the pathologic diagnosis of CoCC. Komuta et al. proposed that CoCC is defined as when the proportion of CoCC accounts for more than 90% of the entire tumor [8]. According to this strict definition, CoCC with a significant proportion of ICC or HCC components may be considered to be an ordinary ICC or HCC, respectively [4]. The authors also reported immunohistochemical findings of a positive EMA at the apical membrane [6, 8, 9], positive NCAM (a marker of hepatic progenitor cells), and negative S100P (a marker of intrahepatic large bile ducts) [8]. As described, we made an incorrect initial diagnosis of ordinary ICC in the present patient, primarily because a lack of knowledge of CoCC led us not to consider the possibility of CoCC. There may be a concern that pathological diagnosis of the recurrent lesions treated with RFA was not made. Therefore, de novo HCC cannot be absolutely ruled out. However, recurrence with a short interval and no underlying liver disease (chronic hepatitis and fatty liver) highly suggest CoCC, rather than de novo HCC.

The present patient has survived for 9.2 years without disease despite eight hepatic recurrences and tumor exposure during the second hepatectomy. This favorable outcome was explained by a liver-limited relapse, small number and size of the recurrent tumors, and no local relapse at the positive surgical margin. These results were due to the less aggressive nature of CoCC compared to ICC [4, 6] and partly because of its overlapping nature with HCC. Another related factor was the aggressive strategy employed for this patient’s treatment. We performed a hepatectomy for the first hepatic recurrence with three tumors and adjuvant chemotherapy using gemcitabine according to the typical strategy for recurrent ICC [10]. However, a subsequent liver recurrence was found immediately after completion of the planned chemotherapy. This fact suggests that systemic chemotherapy may be not effective for CoCC due to its different cell lineage.

To our knowledge, besides the present case, there are three English case reports discussing local treatment for intrahepatic recurrence of CoCC (Table 2) [11–13]. The initial disease-free intervals ranged from 7 months to 3 years. One patient died of disease 13 months after surgery [11], another patient underwent hepatectomy and survived for 4 years without recurrence [12], and the other patient underwent six sessions of hepatectomy and survived for 7 years [13]. The present patient underwent hepatectomy and seven percutaneous RFAs and has survived for more than 8 years despite several recurrences. These findings strongly suggest that localized treatment may be a promising approach for achieving long-term survival.

**Table 2:** Reported cases of cholangiolocellular carcinoma with intrahepatic recurrences controlled by local treatment

Reference	Age (years), sex	Size of primary tumor (cm)	Interval of initial recurrence (months)	No. of disease recurrence	Treatment for recurrences	Follow-up (month)^*^
Maeda et al. [11]	68, M	3	7	1	1 hepatectomy	13, DOD
Yamamoto et al. [12]	61, F	Not available	36	1	1 hepatectomy	48, NED
Tomioku et al. [13]	59, F	10	18	6	6 hepatectomies	84, NED
Present case	40, M	13	10	8	1 hepatectomy and 7 RFAs	110, NED

*F* female, *M* male, *RFA* radiofrequency ablation, *NED* no evidence of disease, *DOD* died of disease

^*^after resection for primary lesion

RFA is a widely applicable treatment for hepatic malignancies [14], particularly in unresectable carcinomas. A few studies and one meta-analysis demonstrated the usefulness of RFA for ICC [15–17]. A large multi-institutional study, however, failed to show the benefit of local treatments compared to systematic chemotherapy; the median survival times were 18.0 and 16.8 months, respectively [18]. Therefore, RFA for unresectable ICC has been controversial. Transcatheter arterial chemoembolization (TACE) is another potential option for unresectable ICC [18]. The tumor in the present patient exhibited enhancement in the arterial phase, which persisted until delayed phase. This CT finding indicates less much cellularity with fibrosis and precludes an efficient local control by TACE. Therefore, we performed RFA for the treatment of recurrent ICC, although our first-line approach is surgical resection when technically feasible. Further, systemic chemotherapy may be considered when the recurrent lesion is not amenable to these local treatments.

Some factors led to successful RFA in the current patient. First, CoCC presented as a hepatic parenchymal mass without periductal and vascular invasions [6]. These longitudinal growth patterns [19], if present, are not amenable to RFA. Second, intensive follow-up using MRI every 1 to 2 months enabled early detection of liver recurrences that were small in size and of a limited number, as shown in Table 1. Early detection is very important for RFA because large and multiple tumors are associated with poor prognosis [14]. Meanwhile, such intensive follow-up has a disadvantage in terms of cost and time effectiveness. To the best of our knowledge, this is the first study to demonstrate a satisfactory outcome for CoCC after RFA. RFA may be a useful option for limited hepatic recurrences in CoCC.

## Conclusions

This case report demonstrated the effectiveness of localized treatment for hepatic recurrences and a difficult CoCC diagnosis. Due to the less aggressive nature of CoCC, liver-targeting localized therapies benefit patients with liver recurrences of CoCC. RFA can be considered to be a therapeutic option for limited hepatic recurrence of CoCC.

## Abbreviations

AFP: Alpha-fetoprotein
CA19-9: Carbohydrate antigen 19-9
CoCC: Cholangiolocellular carcinoma
CT: Computed tomography
EMA: Epithelial membrane antigen
EOB-MRI: Gadoxetic acid-enhanced magnetic resonance imaging
HCC: Hepatocellular carcinoma
ICC: Intrahepatic cholangiocarcinoma
MRI: Magnetic resonance imaging
NCAM: Neural cell adhesion molecule
PIVKA-II: Protein induced by vitamin K absence or antagonist-II
RFA: Radiofrequency ablation
TACE: Transcatheter arterial chemoembolization
